# Examining the Workability, Mechanical, and Thermal Characteristics of Eco-Friendly, Structural Self-Compacting Lightweight Concrete Enhanced with Fly Ash and Silica Fume

**DOI:** 10.3390/ma17143504

**Published:** 2024-07-15

**Authors:** Zehra Funda Akbulut, Demet Yavuz, Taher A. Tawfik, Piotr Smarzewski, Soner Guler

**Affiliations:** 1 Department of Mining Engineering, Faculty of Engineering, University of Van Yüzüncü Yıl, Van 65080, Turkey; 2 Department of Civil Engineering, Faculty of Engineering, University of Van Yüzüncü Yıl, Van 65080, Turkey; demetyavuz@yyu.edu.tr (D.Y.); gulersoner@yyu.edu.tr (S.G.); 3 Institute of Construction and Architecture, Slovak Academy of Sciences, Dúbravská cesta 9, SK-845 03 Bratislava, Slovakia; dr.taher_tawfik@csi.edu.eg; 4 Department of Construction and Building Engineering, High Institute of Engineering, October 6 City 12585, Egypt; 5 Faculty of Civil Engineering and Geodesy, Military University of Technology, 00-908 Warsaw, Poland

**Keywords:** structural self-compacting lightweight concrete, pumice aggregate, expanded perlite aggregate, silica fume, fly ash

## Abstract

This study compares the workability, mechanical, and thermal characteristics of structural self-compacting lightweight concrete (SCLWC) formulations using pumice aggregate (PA), expanded perlite aggregate (EPA), fly ash (FA), and silica fume (SF). FA and SF were used as partial substitutes for cement at a 10% ratio in various mixes, impacting different aspects: According to the obtained results, FA enhanced the workability but SF reduced it, while SF improved the compressive and splitting tensile strengths more than FA. EPA, used as a fine aggregate alongside PA, decreased the workability, compressive strength, and splitting tensile strength compared to the control mix (K0). The thermal properties were altered by FA and SF similarly, while EPA notably reduced the thermal conductivity coefficients. The thermal conductivity coefficients (TCCs) of the K0–K4 SCLWC mixtures ranged from 0.275 to 0.364 W/mK. K0 had a TCC of 0.364 W/mK. With 10% FA, K1 achieved 0.305 W/mK; K2 with 10% SF reached 0.325 W/mK. K3 and K4, using EPA instead of PA, showed significantly lower TCC values: 0.275 W/mK and 0.289 W/mK, respectively. FA and SF improved the thermal conductivity compared to K0, while EPA further reduced the TCC values in K3 and K4 compared to K1 and K2. The compressive strength (CS) values of the K0–K4 SCLWC mixtures at 7 and 28 days reveal notable trends. Using 10% FA in K1 decreased the CS at both 7 days (12.16 MPa) and 28 days (22.36 MPa), attributed to FA’s gradual pozzolanic activity. Conversely, K2 with SF showed increased CS at 7 days (17.88 MPa) and 28 days (29.89 MPa) due to SF’s rapid pozzolanic activity. Incorporating EPA into K3 and K4 reduced the CS values compared to PA, indicating EPA’s lower strength contribution due to its porous structure.

## 1. Introduction

Concrete is one of the fundamental building materials in the construction sector. Modern structures’ durability, reliability, and performance often depend on concrete’s qualities [1,2,3]. However, conventional concrete mixtures may not always provide the desired properties or meet specific requirements. Hence, different types of concrete have been developed in the construction industry, including self-compacting concrete (SCC). SCC belongs to the concrete class with a low water-to-cement ratio, offering high strength and durability [4,5]. These properties ensure that structures are strong and resilient. Among the critical characteristics of SCC are its ability to fill molds and high resistance to segregation [6]. These attributes are achieved through techniques such as superplasticizers and increasing the number of fine materials. However, SCC has disadvantages, such as a high unit weight, which increases the weight and costs of structures [7]. To overcome these issues, using lightweight aggregates emerges as a significant solution. Utilizing lightweight aggregates can reduce the weight of structures, enabling them to experience less oscillation during earthquakes and thus enhancing their earthquake resistance [8,9]. Natural lightweight aggregates like pumice aggregate (PA) and perlite aggregate are abundant, especially in the Eastern Anatolia region of Turkey [10]. They can be used in SCC mixtures to produce lightweight and cost-effective structural self-compacting lightweight concrete (SCLWC).

Additionally, incorporating pumice aggregate (PA) and expanded perlite aggregate (EPA) in SCLWC can enhance its thermal insulation properties and contribute to energy efficiency [11]. SCLWC, a specialized type of concrete, has been increasingly used for structural purposes in recent years [12]. It is mainly employed to reduce the dead load of structures and the weights of reinforced concrete elements used. Lightweight aggregates that contain air voids improve important characteristics of concrete, such as giving it a higher strength-to-weight ratio, better resistance to cracking, less thermal expansion, and superior thermal and sound insulation properties. Additionally, they are beneficial for constructing tall buildings over large areas and longer spans [13]. The most common method for producing SCLWC is using lightweight aggregates with voids. Researchers have extensively studied the effect of aggregate properties on concrete quality. Chen et al. [14] demonstrated that the compressive strength of concrete is primarily influenced by aggregate volume and properties. Yehia et al. [15] indicated that the compressive strength of concrete varies depending on the type of aggregate used, and it increases with concrete density. Agrawal et al. [16], as well as Evangelista et al. [17], reported in their studies that concrete produced with lightweight aggregates has a higher strength-to-weight ratio, better resistance to splitting, a lower coefficient of thermal expansion, and superior thermal and sound insulation properties due to the air voids in these aggregates.

Furthermore, Sahoo et al. [18] revealed that using lightweight aggregates in concrete reduces structures’ dead load and the reinforcement required. In addition to mechanical and thermal properties, the use of different types of pozzolanic materials in the production of SCLWC has been relatively limited. Ibrahim et al. [19] found that hardened concrete samples exhibited lower thermal conductivity when expanded perlite aggregate was used instead of pumice in concrete samples. Türkmen and Gavgalı [20] discovered that concrete made with 10% silica fume (SF) +20% blast furnace slag instead of Portland cement had the lowest permeability coefficient compared to regular concrete. Liu et al. [21] indicated that increasing the use of expanded perlite aggregate (EPA) in concrete resulted in decreased thermal conductivity values, thereby improving the thermal properties of concrete. They also observed similar decreases in the unit volume weights of samples, suggesting a significant reduction in the dead load of structures. However, they noted that the addition of EPA to the mixtures led to a decrease in compressive and flexural strengths and an increase in the water absorption properties of the samples.

This study aims to compare the workability, mechanical, and thermal properties of five different SCLWC mixtures produced with lightweight aggregates such as pumice aggregate (PA), expanded perlite aggregate (EPA), and pozzolanic materials such as fly ash (FA) and silica fume (SF). The workability properties of fresh SCLWC, such as the slump flow diameter, L-box height ratio, and V-funnel discharge time, are evaluated and compared. The mechanical properties of SCLWC specimens, including compressive strength and splitting tensile strength, are determined at 7 and 28 days and compared. The thermal properties of SCLWC specimens are evaluated by obtaining the thermal conductivity coefficients of 28-day specimens and comparing them.

In conclusion, this study illuminates how various lightweight aggregates and pozzolanic materials influence the properties of structural lightweight self-compacting concrete (SCLWC). These findings are pivotal for advancing concrete mixtures that ensure safer, more durable, and environmentally friendly structures within the construction industry. The systematic investigation conducted here rigorously examines the impact of lightweight aggregates and pozzolanic materials on the workability, mechanical strength, and thermal properties of SCLWC. By providing a comprehensive analysis of their contributions, particularly in terms of enhancing structural strength, energy efficiency, and sustainability, this research addresses critical gaps in the current literature. It opens new avenues for developing sophisticated concrete formulations that bolster structural resilience, promote energy efficiency, and support environmental sustainability in structural engineering practices.

## 2. Experimental Program

### 2.1. Materials Used in the SCLWC Mixtures

**Figure 1:**
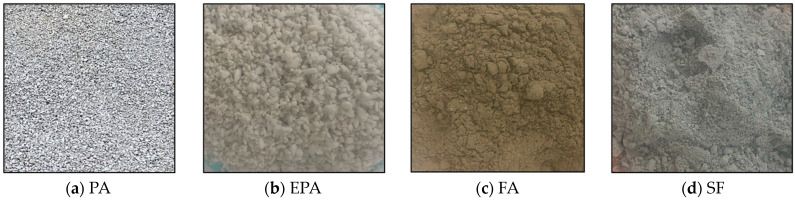
The view of the PA, EPA, FA, and SF.

**Figure 2:**
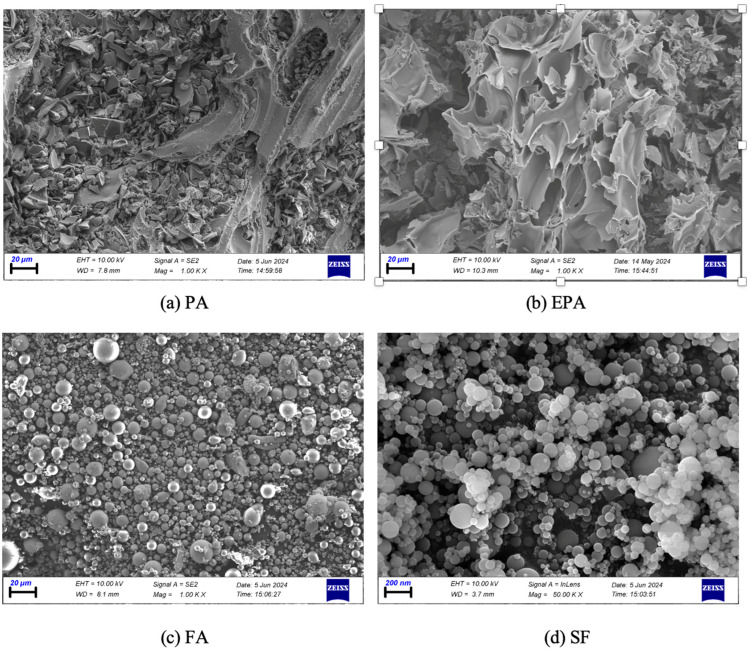
The SEM images of PA, EPA, FA, and SF.

In preparing the SCLWC mixtures, CEM I 42.5 R Portland Cement produced in accordance with the TS EN 197-1 [22] standard by the Van Cement Factory was used. For the SCLWC mixtures, pumice aggregate (PA) with particle sizes of 0–4 mm, 4–8 mm, and 8–16 mm were used as fine aggregates, and expanded perlite aggregate (EPA) with a particle size of only 0–4 mm was used. Fly ash (FA) and silica fume (SF) were used as pozzolanic materials. F-type FA material was obtained from İsken Su Gözü thermal power plant. The silica fume (SF) used in this study was obtained through online sales from Eti Electrometallurgy A.Ş. facilities in Antalya, Turkey. MasterGlenium 51 superplasticizer (SP) additive was used to ensure suitable workability in all mixtures. In the SCLWC mixtures containing PA, EPA, and FA additives, SP was used at a concentration of 0.1% of the total binder content by weight. However, in the K2 and K4 mixtures containing silica fume (SF), SP was used at 0.2% of the binder content by weight to reduce its adverse effects on workability. Images of the PA, EPA, FA, and SF used in the SCLWC mixtures are shown in Figure 1. The interior images of PA, EPA, FA, and SF were obtained with a scanning electron microscope (SEM) analysis under ambient conditions, as shown in Figure 2. Table 1 illustrates the technical characteristics of PC, FA, SF, PA, and EPA.

### 2.2. Mixing Phases of SCLWC Mixtures

A concrete mixer was used to prepare the SCLWC mixtures. Initially, in the preparation of the SCLWC mixtures, Portland cement (PC), pumice aggregate (PA), expanded perlite aggregates (EPAs), silica fume (SF), and fly ash (FA) were dry mixed in a concrete mixer for 2 min. Then, half of the required water and the superplasticizer (SP) additive was added to the dry mixture and mixed for 3 min. Thus, each mixture was mixed for a total of 5 min. Following the mixing process, fresh SCLWC mixtures underwent slump flow, L-box, and V-funnel tests according to the EFNARC standard [23]. In the second part of the study, the thermal conductivity coefficients (TCCs), compressive strengths (CSs), and splitting tensile strengths (STSs) of hardened SCLWC samples were determined. The CSs and STSs of the SCLWC samples were determined using cube specimens with a side length of 150 mm. For the determination of the thermal conductivity coefficients of the SCLWC samples, plates with dimensions of 30 cm × 30 cm × 3 cm were used. The produced SCLWC samples were kept in molds in ambient conditions for 24 h. At the end of this period, the samples were removed from the molds and cured in a curing tank at a temperature of 20 ± 2 °C for 28 days. In all experiments, three samples were tested for each mixture, and their averages were taken. The material quantities that were used in SCLWC mixtures are given in Table 2.

### 2.3. Fresh and Hardened Tests of SCLWC Mixtures

Firstly, the fresh density (FRD) of the SCLWC samples was measured while they were fresh. Subsequently, after 24 h, the samples were removed from the molds and placed in an oven at 105 °C for one day. Following this period, their hardened densities (HDs) were measured. To determine the workability of fresh SCLWC mixes, slump flow, L-box, and V-funnel tests were carried out according to the EFNARC standard [23]. In the slump flow test, the SCLWC mixtures were poured into an Abrams cone without undergoing any compaction process, and after removing the cone, the total flow dimeters (FDs) in two perpendicular directions were measured, and their averages were taken. The V-funnel apparatus was used to determine the viscosity properties of the SCLWC mixes. After filling the V-funnel with fresh concrete, the time taken for the concrete to empty through the bottom cap was measured to assess the flowability of the mix. The standard L-box apparatus measured the passing ability of SCLWC mixes. Immediately after the SCLWC mixing process was completed, concrete was filled into the closed section of the L-box without any waiting or vibration processes. Following filling, the lid was pulled upward at a constant speed, and the passing ability of the concrete through the steel bars was determined through the ratio between the elevations of the two end points. The acceptance criteria for self-compacting concrete according to the EFNARC standards are provided in Table 3.

Heat conductivity measurements were performed on SCLWC slab specimens with dimensions of 30 cm × 30 cm × 3 cm. The KEM QTM-500 test device, which has a measurement range of 0.023–11.63 W/mK, was utilized for insulation tests. Due to the inability of the experimental device to measure moist materials, the measurements were conducted on specimens dried in an oven following the EN 1745 standard [24]. The obtained results were taken as the arithmetic average of the three experiments. The compressive strength (CS) and splitting tensile strength (STS) of the SCLWC specimens at 7 and 28 days were determined by the TS EN 12390-3 [25] and TS EN 12390-6 [26] standards, respectively. Cubic specimens with a side length of 70 mm and 150 mm were used as samples for the determination of CS and STS, respectively. The CS and STS values of the SCLWC specimens were calculated by taking the arithmetic average of the three specimens broken for each experiment.

## 3. Results and Discussion

### 3.1. Workability of SCLWC Mixtures

#### 3.1.1. Density and Slump Flow of SCLWC Mixtures

**Figure 3:**
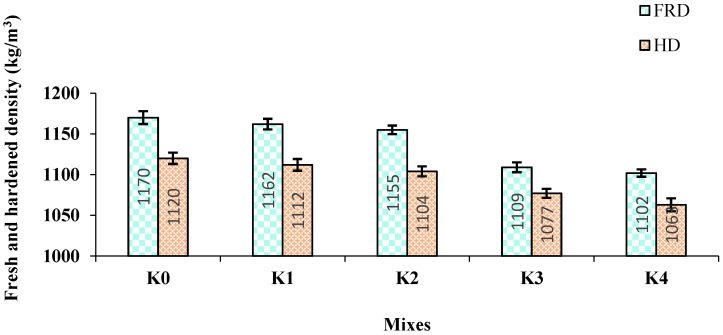
Fresh density (FRD) and hardened density (HD) results for SCLWC samples.

The fresh density (FRD) and hardened density (HD) results for the SCLWC samples are given in Figure 3.

**Figure 4:**
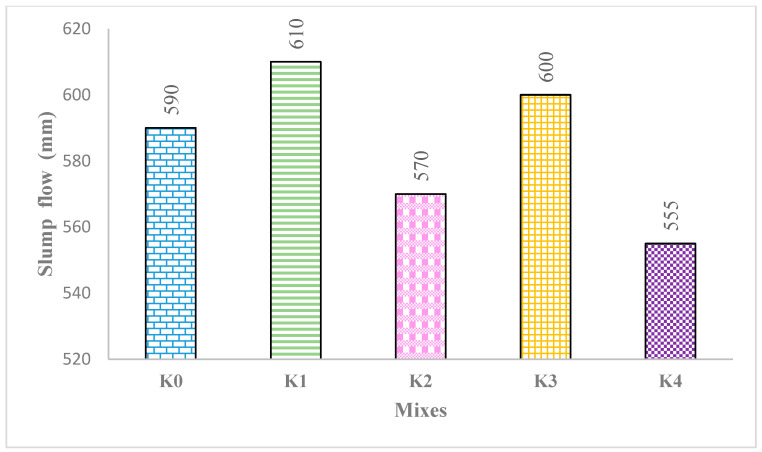
The slump flow of the K0–K4 SCLWC mixtures.

The slump flows of the K0–K4 SCLWCs are shown in Figure 4. The slump flow diameters of all the mixtures produced on the slump flow table, where the fluidity and resistance to segregation of the self-compacting structural lightweight concrete (SCLWC) mixtures were measured, varied between 555 mm and 610 mm. A slump flow diameter of 590 mm was achieved for the control mixture, K0. When 10% fly ash (FA) was used in mixture K1, a slump flow diameter of 610 mm was obtained, while in mixture K2 with 10% silica fume (SF), the slump flow diameter slightly decreased to 570 mm. In mixtures K3 and K4, where expanded perlite aggregate (EPA) was used instead of pumice aggregate (PA) with a 0–4 mm size, slump flow diameters of 600 mm and 555 mm were obtained, respectively. These values were found to meet the requirements of the SF1 class conditions defined by the EFNARC standard for self-compacting concrete. As evident from these results, using 10% FA in mixtures positively affected the workability of the mixtures, resulting in an increase in the slump flow diameter of fresh SCLWC compared to the K0 control. A significant reason for this is FA’s glassy and spherical particle structure [27]. However, substituting cement with 10% SF adversely affected the workability of the SCLWC mixtures, thus causing slightly lower slump flow diameters compared to the K0 and K1 control mixtures. To mitigate SF’s negative impact on SCLWC’s workability, a superplasticizer (SP) additive was used at twice the rate of other mixtures in SF-modified SCLWC. SF primarily reduces SCLWC’s workability due to its high surface area, leading to water absorption and decreased workability [28]. Using EPA instead of PA negatively affected the workability of the mixtures [29], resulting in slightly lower slump flow diameters for mixtures K3 and K4 than the K0 control mixture.

#### 3.1.2. L-Box of SCLWC Mixtures

**Figure 5:**
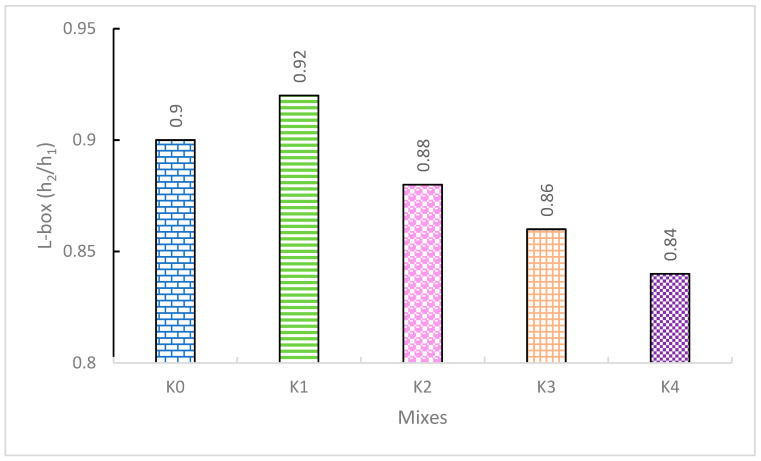
The L-box values of the K0–K4 SCLWC mixtures.

The ratio of the two height levels in the flow region, denoted as h_2_/h_1_, was obtained for all SCLWC mixtures using the L-box apparatus to evaluate their passing ability. The L-box apparatus is employed in measuring passing ability in self-compacting fresh concrete mixtures [30,31,32]. As observed in Figure 5, the L-box measurements of the SCLWC mixtures ranged between 0.84 and 0.92. In the K0 control mixture, the h_2_/h_1_ ratio was obtained as 0.90. For the K1 mixture with 10% FA, the h_2_/h_1_ ratio was 0.92, while for the K2 mixture with 10% SF, the h_2_/h_1_ ratio slightly decreased to 0.88. In mixtures K3 and K4, where EPA was used instead of 0–4 mm PA, the h_2_/h_1_ ratio decreased slightly to 0.86 and 0.84, respectively. As seen from Figure 4, the K0–K4 SCLWC mixtures all met the acceptable limit value of 0.8 defined by the EFNARC standard. Therefore, it can be understood from these results that using different types of aggregates and pozzolanic materials did not cause a significant change in the passing ability of the SCLWC mixtures. However, compared to the K0 control mixture, using FA increased the fluidity of the SCLWC mixtures and enhanced their passing abilities slightly [33]. However, the use of SF and EPA reduced the fluidity of the SCLWC mixtures due to their excessive water absorption, resulting in a slight decrease in the h_2_/h_1_ ratio of the SCLWC fresh mixtures [34,35].

#### 3.1.3. V-Funnel of SCLWC Mixtures

**Figure 6:**
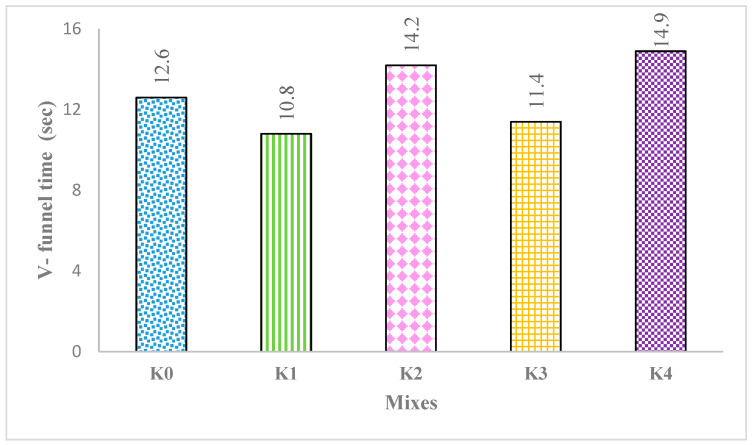
The V-funnel times of the K0–K4 SCLWC mixtures.

The V-funnel test is a method used to assess the filling ability and resistance to segregation of SCLWC mixtures. The discharge times in the V-funnel, which enable the evaluation of the workability and viscosity of SCLWC mixtures, are provided in Figure 6. As seen in Figure 6, the V-funnel measurements of the SCLWC mixtures varied between 10.8 s and 14.9 s. In the K0 control mixture, the V-funnel discharge time was 12.6 s. For the K1 mixture with 10% FA, the V-funnel discharge time was 10.8 s, while for the K2 mixture with 10% SF, the V-funnel discharge time slightly increased to 14.2 s. Comparing mixtures K1 and K2, in mixtures K3 and K4, where EPA was used instead of 0–4 mm PA, the V-funnel discharge time increased slightly to 11.4 s and 14.9 s, respectively. Figure 5 shows that all the K0–K4 SCLWC mixtures met the acceptable VF2 limit values defined by the EFNARC standard, which range from 9 to 27 s. As shown in Figure 6, compared to the K0 control mixture, using FA in the SCLWC mixtures increased the fluidity and filling ability of the mixtures, resulting in shorter V-funnel discharge times. In contrast, using SF and EPA in the SCLWC mixtures reduced the fluidity, increased the viscosity of the SCLWC mixtures, and consequently led to higher V-funnel discharge times.

#### 3.1.4. Thermal Conductivity Coefficient

**Figure 7:**
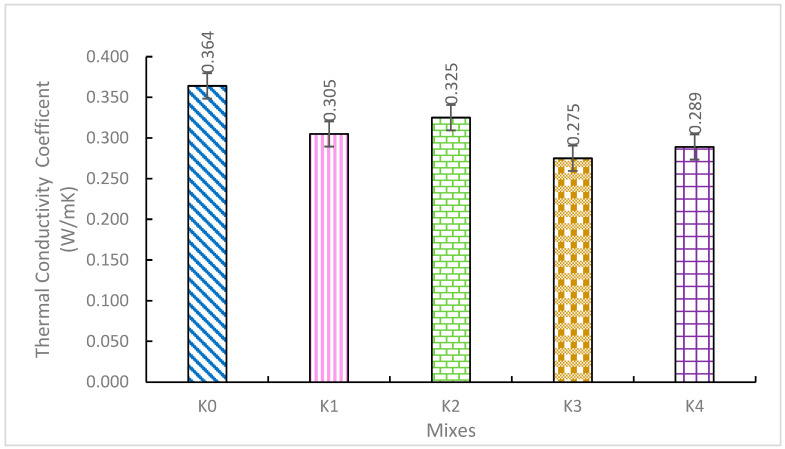
Thermal conductivity coefficients (TCCs) of SCLWC mixtures.

The thermal conductivity coefficients (TCCs) of the K0–K4 SCLWC mixtures are provided in Figure 7. As depicted in Figure 7, the TCC values of the SCLWC mixtures ranged between 0.275 and 0.364 W/mK. The TCC value of the K0 control mixture was obtained as 0.364 W/mK. For the K1 mixture with 10% FA, the TCC value was 0.305 W/mK; for the K2 mixture with 10% SF, the TCC value was also 0.325 W/mK. Comparing mixtures K1 and K2, in mixtures K3 and K4 where EPA was used instead of 0–4 mm PA, the TCC values decreased significantly to 0.275 W/mK and 0.289 W/mK, respectively. As seen in Figure 6, compared to the K0 control mixture, the use of FA and SF in the SCLWC mixtures improved their thermal conductivity properties, resulting in lower TCC values for the K1-K4 mixtures. Additionally, in samples K3 and K4, where EPA was used instead of PA, the thermal conductivity properties improved significantly, resulting in lower TCC values compared to samples K1 and K2. Since the density of EPA is considerably lower than that of other building materials (such as cement, aggregate, etc.), it plays a crucial role in reducing dead loads in structures and improving the thermal properties of the produced material. EPA’s low thermal conductivity enhances the produced concrete’s thermal properties [36,37].

#### 3.1.5. Compressive Strength

**Figure 8:**
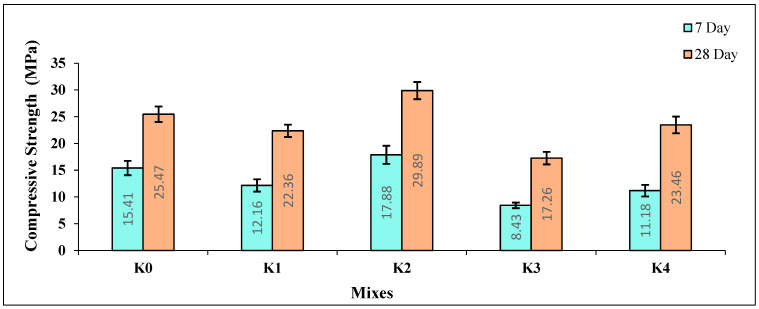
The 7- and 28-day compressive strength (CS) values of the SCLWC mixtures.

The compressive strength (CS) values of the K0–K4 SCLWC mixtures at 7 and 28 days are presented in Figure 8. Upon examining the 7-day CS of the SCLWC samples, it is observed that the CS value of the K0 control sample is 15.41 MPa. The CS value of the K1 sample with FA slightly decreases to 12.16 MPa, while the CS value of the K2 sample with SF slightly increases to 17.88 MPa. The CS value of the K3 sample with FA and EPA decreases compared to the K1 sample to 8.43 MPa. Similarly, the CS value of the K4 sample with SF and EPA decreases compared to the K2 sample to 11.18 MPa. When examining the 28-day CS values of the SCLWC samples, it is observed that the CS value of the K0 control sample is 25.47 MPa. The CS value of the K1 sample with FA slightly decreases to 22.36 MPa, while the CS value of the K2 sample with SF slightly increases to 29.89 MPa. The CS value of the K3 sample with FA and EPA decreases somewhat compared to the K1 sample to 17.26 MPa.

Similarly, the CS value of the K4 sample with SF and EPA decreases compared to the K2 sample to 23.46 MPa. As is known, FA and SF are pozzolanic materials that form calcium silicate hydrate (C-S-H) bonds in the concrete matrix, which provide additional strength by reacting with portlandite (CH), a hydration product of cement [38,39]. As seen in Figure 8, compared to the K0 control sample, using 10% FA in the mixtures, replacing part of the cement, as in the K1 samples, led to a decrease in both the 7-day and 28-day CS values. The main reason for this is attributed to the pozzolanic activity of FA progressing over time and showing its effect over a more extended period [40]. In contrast, the use of SF in the mixtures, as in the K2 sample, increased both the 7-day and 28-day CS values compared to the K0 control sample. The main reason for this is the faster pozzolanic activity of SF, which plays a more influential role in improving both the 7-day and 28-day CS values compared to FA. Using EPA instead of PA in the mixtures decreased the CS values of both the FA- and SF-modified samples, K2 and K4. This is due to the more porous and sponge-like structure of EPA compared to PA, resulting in a decrease in the CS values of the K2 and K4 samples.

#### 3.1.6. Splitting Tensile Strength

**Figure 9:**
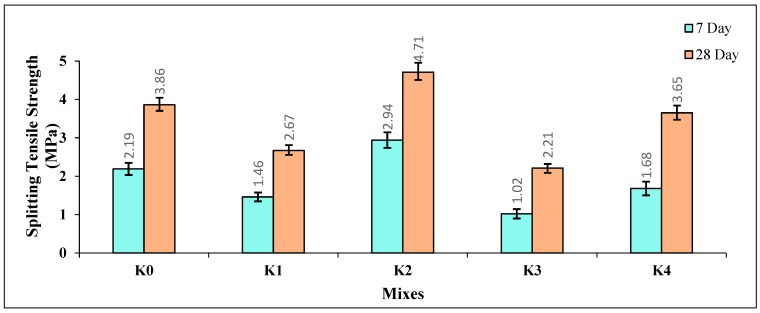
The 7- and 28-day splitting tensile strength (STS) values of the SCLWC mixtures.

The splitting tensile strength (STS) values of the K0–K4 SCLWC mixtures at 7 and 28 days are provided in Figure 9. When examining the 7-day STS values of the SCLWC samples, it is observed that the STS value of the K0 control sample is 2.19 MPa. The STS value of the K1 sample with FA slightly decreases to 1.46 MPa, while the STS value of the K2 sample with SF slightly increases to 2.94 MPa. The STS value of the K3 sample with FA and EPA decreases compared to the K1 sample to 1.02 MPa. Similarly, the STS value of the K4 sample with SF and EPA decreases compared to the K2 sample to 1.68 MPa. When examining the 28-day STS values of the SCLWC samples, it is observed that the STS value of the K0 control sample is 3.86 MPa. The STS value of the K1 sample with FA slightly decreases to 2.67 MPa, while the STS value of the K2 sample with SF slightly increases to 4.71 MPa. The STS value of the K3 sample with FA and EPA decreases slightly compared to the K1 sample to 2.21 MPa. Similarly, the STS value of the K4 sample with SF and EPA decreases compared to the K2 sample to 3.65 MPa.

As seen in Figure 9, the trend observed in the CS values of the SCLWC samples is also evident in the STS values. The pozzolanic materials FA and SF were expected to improve the STS values of the SCLWC samples by forming calcium-silicate-hydrate (C-S-H) bonds, providing additional strength to the concrete matrix [41]. However, as seen from Figure 8, compared to the K0 control sample, using 10% FA in the mixtures, replacing part of the cement, as in the K1 samples, led to a decrease in both the 7-day and 28-day STS values. Like the CS values, the main reason for this can be attributed to the pozzolanic activity of FA progressing over time and showing its effect over a more extended period. In contrast, the use of SF in the mixtures, as in the K2 sample, increased both the 7-day and 28-day STS values compared to the K0 control sample. The main reason for this is the faster pozzolanic activity of SF, which plays a more influential role in improving both the 7-day and 28-day STS values compared to FA. Using EPA instead of PA in the mixtures decreased the STS values of both the FA- and SF-modified samples, K2 and K4. This is due to the more porous and sponge-like structure of EPA compared to PA, resulting in a decrease in the STS values of the K2 and K4 samples.

#### 3.1.7. SEM Analysis

**Figure 10:**
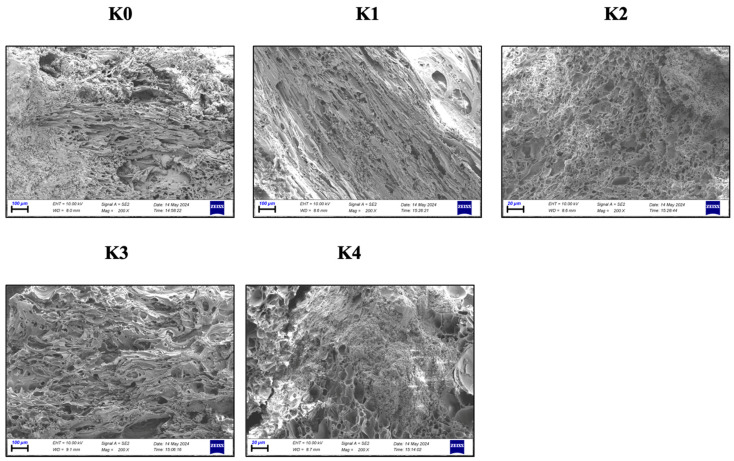
The SEM analysis images of the K0–K4 SCLWC specimens.

The SEM analysis images of the K0–K4 SCLWC specimens are provided in Figure 10. Upon examination of Figure 10, the K0 control specimen exhibits macropores and an irregular internal structure. This irregular and porous cellular structure contributes to the SCLWC specimens being permeable and lacking strength [42]. When the SEM images are analyzed, the SCLWC specimens with 10% FA and SF additives have smaller and more regular pores than the K0 reference specimen. It is observed that FA and SF fill the voids in the SCLWC specimen matrix, making the internal structure more regular. Replacing PA voids with FA and SF and the reactions occurring during production transform the material structure from a macroporous state to a microporous one [43]. As shown in Figure 10, the K0 control specimen has more macrovoids and capillary cracks than the K1–K4 specimens.

Moreover, partially or entirely unreacted spherical FA particles can be observed in the SEM images of samples K1 and K3. Weak interfacial transition zones (ITZs) between cement and PA in the K0 control specimen are reinforced with the filling effect of the FA and SF pozzolanic materials in samples K1 and K3. The substitution of cement with finer particles of FA and SF renders the ITZ regions of the SCLWC matrix more impermeable and robust. Additionally, the formation of new calcium-silicate-hydrate (C-S-H) bonds in the matrix, mainly due to the higher pozzolanic activity of SF, contributes to the improvement in both the 7-day and 28-day compressive strength (CS) and splitting tensile strength (STS) properties. The SEM images of the K3 and K4 specimens with EPA enable a detailed examination of the internal structure of the SCLWC matrix. In these images, it is observed that the SCLWC matrix exhibits a homogeneous structure with regularly embedded EPA particles. EPA’s smooth and rounded structure contributes to reducing pores within the concrete matrix, thereby enhancing the SCLWC matrix’s properties [44].

## 4. Conclusions

This study presents a comprehensive comparative analysis of the workability, mechanical, and thermal properties of structural self-compacting lightweight concretes (SCLWCs) fabricated utilizing varying combinations of constituent materials, including pumice aggregate (PA), expanded perlite aggregate (EPA), fly ash (FA), and silica fume (SF). The investigation encompassed five distinct SCLWC formulations, except for the K0 control mixture.

- The incorporation of fly ash (FA) and silica fume (SF) into structural self-compacting lightweight concrete (SCLWC) showed significant effects on both its workability and mechanical properties. Fly ash was observed to enhance workability, whereas silica fume, while decreasing workability, was found to improve the compressive strength (CS) and splitting tensile strength (STS) of the SCLWC samples.
- Pumice aggregate (PA) and expanded perlite aggregate (EPA) demonstrated distinct effects on the properties of the SCLWC mixtures. PA, utilized in different sizes across various mixtures, enhanced overall structural integrity. Conversely, EPA, especially when used in conjunction with PA, reduced the workability, compressive strength (CS), and splitting tensile strength (STS) of the SCLWC samples.
- Workability parameters such as the slump flow diameter, the flow time in the V-funnel, and the height ratio in the L-box were influenced differently by the composition of the SCLWC mixtures. Fly ash (FA) improved workability, whereas silica fume (SF) had a detrimental effect, highlighting the importance of the precise selection and proportioning of supplementary materials.
- The selection of aggregates and supplementary materials had a substantial impact on the thermal properties of the SCLWC samples. Expanded perlite aggregate (EPA) notably decreased the thermal conductivity coefficient (TCC), suggesting its potential to enhance thermal insulation properties in construction applications.
- This study emphasizes the significance of understanding material interactions for developing optimized SCLWC mixtures. Achieving a balance between workability, mechanical strength, and thermal performance necessitates careful consideration of aggregate types, supplementary materials, and their proportions.
- In terms of sustainability, this study makes significant contributions towards reducing environmental impacts. Lightweight aggregates like pumice (PA) and expanded perlite (EPA) can minimize natural resource consumption and lower the carbon footprint of building material production. Additionally, the utilization of by-products such as fly ash (FA) and silica fume (SF) represents a crucial step towards recycling waste materials and reducing environmental burdens. Therefore, the findings of this study provide valuable insights for developing sustainable building materials and promoting environmentally friendly practices in the construction sector.

## Figures and Tables

**Table 1:** Chemical components and physical properties of PC, FA, and SF.

Chemical Properties	PC	FA	SF	PA	EPA
(Component)	(%)	(%)	(%)	(%)	(%)
SiO_2_	20.23	60.32	91.44	75.2	70.64
Al_2_O_3_	5.33	23.87	1.78	14.1	13.01
Fe_2_O_3_	4.01	7.62	0.45	2.47	1.02
CaO	62.55	2.03	0.28	1.30	2.01
MgO	2.81	2.05	0.08	0.03	1.74
Na_2_O	0.22	0.26	1.97	0.2	3.57
K_2_O	0.91	0.37	0.22	5.25	4.37
Loss of ignition (%)	2.94	-	-	3.30	-
Density (g/cm^3^)	2.95	2.04	2.32	-	0.08

**Table 2:** Mix design of the SCLWC mixtures (kg/m^3^).

Mixes	Mix Code	Cement	FA	SF	(0–4 mm) (PA)	(4–8 mm) (PA)	(8–16 mm) (PA)	(0–4 mm) (EPA)	SP (%)	w/c
Control	K0	400	-	-	450	150	130	-	0.1	0.55
FA_10%	K1	360	40	-	450	150	130	-	0.1	0.55
SF_10%	K2	360	-	40	450	150	130	-	0.2	0.55
FA_10%	K3	360	40	-	-	150	130	450	0.1	0.55
SF_10%	K4	360	-	40	-	150	130	450	0.2	0.55

**Table 3:** Acceptance criteria for self-compacting concrete according to the EFNARC standards [23].

Experiment	Classification	Range of Values
Slump flow	SF1	55–65 cm
	SF2	66–75 cm
	SF3	76–85 cm
T500 time	VS1	≤2 s
	VS2	>2 s
L-box	PA1	≥0.8 (two and three bars)
V-funnel	VF1	≤8 s
	VF2	9–25 s

## Data Availability

The original contributions presented in the study are included in the article, further inquiries can be directed to the corresponding authors.
